# Cathepsin A inhibition attenuates myocardial infarction-induced heart failure on the functional and proteomic levels

**DOI:** 10.1186/s12967-016-0907-8

**Published:** 2016-05-31

**Authors:** Agnese Petrera, Johann Gassenhuber, Sven Ruf, Deepika Gunasekaran, Jennifer Esser, Jasmin Hasmik Shahinian, Thomas Hübschle, Hartmut Rütten, Thorsten Sadowski, Oliver Schilling

**Affiliations:** Institute for Molecular Medicine and Cell Research, University of Freiburg, Stefan Meier Strasse 17, 79104 Freiburg, Germany; Sanofi-Aventis Deutschland GmbH, Industriepark Höchst, 65926 Frankfurt Am Main, Germany; Department of Cardiology and Angiology, University Heart Center Freiburg, University of Freiburg, Breisacher Strasse 33, 79106 Freiburg, Germany; Department of Cardiac Surgery, University Hospital Basel, Spitalstrasse 21, Basel, Switzerland; BIOSS Centre for Biological Signaling Studies, University of Freiburg, 79104 Freiburg, Germany; German Cancer Consortium (DKTK) and German Cancer Research Center (DKFZ), Heidelberg, Germany

**Keywords:** Cardiovascular diseases, Heart failure, Myocardial infarction, Drug therapy, Mouse model

## Abstract

**Background:**

Myocardial infarction (MI) is a major cause of heart failure. The carboxypeptidase cathepsin A is a novel target in the treatment of cardiac failure. We aim to show that recently developed inhibitors of the protease cathepsin A attenuate post-MI heart failure.

**Methods:**

Mice were subjected to permanent left anterior descending artery (LAD) ligation or sham operation. 24 h post–surgery, LAD-ligated animals were treated with daily doses of the cathepsin A inhibitor SAR1 or placebo. After 4 weeks, the three groups (sham, MI-placebo, MI-SAR1) were evaluated.

**Results:**

Compared to sham-operated animals, placebo-treated mice showed significantly impaired cardiac function and increased plasma BNP levels. Cathepsin A inhibition prevented the increase of plasma BNP levels and displayed a trend towards improved cardiac functionality. Proteomic profiling was performed for the three groups (sham, MI-placebo, MI-SAR1). More than 100 proteins were significantly altered in placebo-treated LAD ligation compared to the sham operation, including known markers of cardiac failure as well as extracellular/matricellular proteins. This ensemble constitutes a proteome fingerprint of myocardial infarction induced by LAD ligation in mice. Cathepsin A inhibitor treatment normalized the marked increase of the muscle stress marker CA3 as well as of Igγ 2b and fatty acid synthase. For numerous further proteins, cathepsin A inhibition partially dampened the LAD ligation-induced proteome alterations.

**Conclusions:**

Our proteomic and functional data suggest that cathepsin A inhibition has cardioprotective properties and support a beneficial effect of cathepsin A inhibition in the treatment of heart failure after myocardial infarction.

**Electronic supplementary material:**

The online version of this article (doi:10.1186/s12967-016-0907-8) contains supplementary material, which is available to authorized users.

## Background

Cathepsin A is a multifunctional protein with diverse sub-cellular localizations. Mostly found in the lysosome, cathepsin A is also present at the cell surface and secreted outside the cell [1, 2]. Cathepsin A is widely known for forming a multi-protein complex with lysosomal neuraminidase and β-galactosidase, which protects β-galactosidase from proteolytic degradation and activates neuraminidase. Genetic deficiency of the cathepsin A protein leads to reduced levels of β-galactosidase and results in the lysosomal storage disease galactosialidosis [1, 2].

Enzymatically, cathepsin A acts as a carboxypeptidase in the lysosome. In mice, expression of catalytically inactive cathepsin A in lieu of the wild-type variant does not cause galactosialidosis, highlighting that cathepsin A enzymatic activity is not linked to its role in protecting β-galactosidase and neuraminidase [3]. In the same publication the authors also report an impact of loss of cathepsin A enzymatic activity on elastic fiber formation and endothelin-1 inactivation. Extracellularly, cathepsin A is involved in the processing of vascular peptides, such as endothelin-1, bradykinin and angiotensin [4].

Cathepsin A has recently gained attention as a promising target for the treatment of heart failure [5]. Cathepsin A mRNA expression is up-regulated in infarcted rat and mouse tissue [6, 7] and its role in the regulation of local bradykinin was demonstrated in animal models of hypertension [8]. Orally available, selective cathepsin A inhibitors have been recently presented, which are based on β-amino acid derivatives [8]. The new inhibitors have beneficial pharmacokinetic profiles and showed remarkable protection in rat models of cardiac hypertrophy and of atrial fibrillation [8]. One of these inhibitors, named SAR1 (refer to as compound 2a in the work of Ruf et al. [8], IC_50_ = 26 nM), successfully passed through phase I clinical trials showing favorable safety profile in healthy young and elderly human subjects [9].

In the last decade, the etiology of heart failure has shifted from hypertension and valvular disease as most common causes to heart failure following myocardial infarction (MI) [10]. MI is defined as a pathological event involving myocardial cell necrosis due to significant and sustained ischemia [11]. MI results from either coronary heart disease, implying obstruction of blood flow due to plaques in the coronary arteries or, much less frequently, to other obstructing mechanisms (e.g. spasm of plaque-free arteries) [11]. In response to MI the left ventricle undergoes profound alterations of its architecture, including interstitial fibrosis, remodeling in the non-infarcted myocardium, and ventricular hypertrophy [12].

Protease inhibition therapy is prominently employed in the area of cardiovascular diseases [13]. Besides anticoagulants, this includes modulators of the renin-angiotensin system, which generally yield vasodilatory effects. In the treatment of post-MI heart failure, vasodilation reduces pre- and afterload and thus displays a cardioprotective effect and reduced cardiac remodeling.

In the present report, we probe the effect of cathepsin A inhibition in a mouse model of LAD ligation using both functional and proteomic analyses.

## Methods

### Animals

Six to seven week old male C57BL/6 mice weighing 19−25 g were purchased from Charles River Laboratories. Twelve weeks old Wistar rats weighing 250–300 g were purchased from Harlan. Animals were housed in an air-conditioned room with a 12 h dark/light cycle and received standard mouse chow with free access to tap water. They were allowed 7 days to adjust to the new environment before starting the experiments. All animal studies conformed to the German law for the protection of animal guidelines and the guide for the care and use of laboratory animals published by the US National Institutes of Health (NIH Publications No 85–23, revised 1996) as well as to Sanofi-Aventis Ethical Committee guidelines.

### Compound characteristics

SAR1 was synthesized in the chemical department of Sanofi. SAR1 ((S)-3-{[1-(2-Fluoro-phenyl)-5-methoxy-1H-pyrazole-3-carbonyl]-amino}-3-o-tolyl-propionic acid) is a new orally active cathepsin A inhibitor previously described as compound 2a by Ruf et al. [8].

### Surgical induction of myocardial infarction

Study animals were randomized to MI versus sham operation. Mice were anaesthetized with 0.1 ml/10 g body weight i.p. of a mixture of ketamine (100 mg/ml), xylazine (20 mg/ml) and atropine (0.5 mg/ml). They were placed on a heated operating table and ventilated at a tidal volume of 7 µl/g body weight. The body temperature was controlled by a rectal probe and maintained at 37 °C. A left thoracotomy was performed via the third intercostal space. MI was induced by placing a 7/0 silk suture around the left anterior descending coronary artery near the atrial auricle. For animals undergoing a sham operation only the chest cavity was opened without further surgical procedures. The lungs were inflated by increasing positive end-expiratory pressure and the thoracotomy site was closed. Animals were allowed to recover and treated with 0.1 mg/10 g body weight buprenorphine (0.3 mg/ml) by subcutaneous injection for post-operative analgesia. Mice were then randomized to placebo or SAR1 (100 mg/kg in chow). Treatment started 1 day after LAD ligation and lasted for 4 weeks. In the SAR1 group fourteen animals died during the 1st week in contrast to eight in the placebo group. No further deaths occurred between weeks two and four in both groups. The difference in mortality under SAR1 treatment did not reach significance (p value >0.08, Mantel-Cox log-rank test). The final sample size at the end of the study for each group was the following: sham (*n* = 10), MI-placebo (*n* = 15), MI-SAR1 (*n* = 10). For determination of infarct size hearts were cut open at the end of the study, photographed and digitized. Images were analyzed for total and the more lucid infarcted area with the Explora Nova image analysis software Morpho Expert (La Rochelle, France) using a Leica- macrosetup.

Surgery in rats was performed in a similar way as described before [14]. They were randomized to placebo or SAR1 (3, 10 and 30 mg/kg, p.o.). Treatment started 1 day after LAD ligation and lasted for 7 days. Sample size for each group was the following: sham (*n* = 10), MI-placebo (*n* = 13), MI-SAR1 3 mg/kg (*n* = 11), MI-SAR1 10 mg/kg (*n* = 11), MI-SAR1 30 mg/kg (*n* = 11).

### Hemodynamic evaluation

For the assessment of pressure–volume relationships, mice were anesthetized with 2 % Isoflurane. Respiration was controlled through a tracheotomy cannula, and animals were mechanically ventilated (Minivent, Hugo Sachs) at 140 breaths/min and a tidal volume of 7 µl room air/g BW. Physiological temperature was maintained with a heated operating table with rectal probe. The left ventricle was catheterized retrogradely via the right carotid artery using a 1.4 F impedance-micromanometer tip catheter (Millar Instruments, Houston, TX, USA). Pressure–volume signals were recorded at steady state and during transient preload reduction achieved by vena cava inferior occlusion. Artificial ventilation was stopped during sampling of approximately ten cardiac cycles for analysis of pressure–volume measurement. Data were digitalized with a sampling rate of 1000 Hz and recorded using specialized software (HEM 3.5, Notocord, France). For subsequent analysis of pressure–volume loops, Notocord and Excel software was used.

In rats hemodynamic parameters were measured in anaesthetized animals using Millar Tip catheters (Millar Instruments Inc, Houston, USA) introduced from the right arteria carotis and advanced into the left ventricle.

### Plasma BNP

Plasma BNP32 was determined with commercially available ELISAs (RayBiotech, Phoenix Pharmaceuticals Inc.)

### Preparation of tissue samples for proteomic analysis

Snap frozen fresh mouse left ventricles (50–100 mg) were lysed in 500 µl of homogenization buffer (100 mM Na-acetate, 5 mM EDTA, 1 mM dithiothreitol, 0.01 mM trans-epoxysuccinyl-L-leucylamido (4-guanidino)butane (E64), 1 mM phenyl-methanesulfonyl fluoride, 0.05 % Brij, pH 5.5) using an Ultra-Turrax and centrifuged at 1000*g* for 15 min at 4 °C. Protein concentrations were determined via BCA protein assay kit (Thermo scientific).

### Quantitative proteome comparison

For proteome comparison, left ventricles from sham operated mice (sham) and mice which underwent left coronary artery ligation treated with placebo or SAR1 were prepared as described above. Preparation of mass spectrometry samples was performed as described previously, including stable isotope labeling with either formaldehyde light [d(0)^12^ C], medium [d(2)^12^ C] or heavy [d(2)^13^ C] for quantitative comparison and pre-fractionation via strong cation exchange chromatography [15]. LC–MS/MS analysis is described in the corresponding section. MS files were analyzed by MaxQuant version 1.3.0.5 with the Uniprot mouse database downloaded on October 2011, counting 44819 entries. MaxQuant analysis included an initial search with a precursor mass tolerance of 20 ppm for mass recalibration. In the main Andromeda search precursor mass and fragment mass were searched with initial mass tolerance of 6 ppm and 0.5 Da respectively. The search included variable modifications of methionine oxidation and N-terminal acetylation, and fixed modification of carbamidomethyl cysteine. Minimal peptide length was set to seven amino acids and a zero missed cleavages were allowed. The false discovery rate (FDR) was set to 0.05 for peptide and protein identifications; however we only considered proteins that were independently identified in at least three replicates. For comparison between samples we used a labeling scheme based on multiplicity three: dimethLys0/dimethNter0 (light label); dimethLys4/dimethNter4 (medium label); dimethLys8/dimethNter8 (heavy label). A minimum of two ratio counts was used to determine the normalized protein intensity. Protein table were filtered to eliminate the identifications from the reverse database, and common contaminants.

### LC–MS/MS analysis

Analysis was performed on an Orbitrap XL (Thermo scientific) mass spectrometer that was coupled to an Ultimate 3000 micro pump (Thermo scientific). Buffer A was 0.5 % acetic acid, buffer B 0.5 % acetic acid in 80 % acetonitrile (HPLC grade). Liquid phases were applied at a flow rate of 300 nl/min with an increasing gradient of organic solvent for peptide separation. Reprosil-Pur 120 ODS-3 (Dr. Maisch, Ammerbuch, Germany) was used to pack column tips of 75 µm inner diameter and 11 cm length. The MS was operated in data dependent mode and each MS scan was followed by a maximum of five MS/MS scans.

### Western blot

30 μg of heart tissue lysate were loaded on to 12 % SDS–polyacrylamide gels. GAPDH served as an internal loading control. After electrophoretic separation, proteins were transferred on polyvinylidene fluoride membranes using a semidry blot system (Bio-Rad, Munich, Germany). After blocking, the membranes were exposed to the primary antibodies (GAPDH, 1:1000; carbonic anhydrase 3, 1:200; periostin, 1:500; troponin T, 1:1000; cytoglobin, 1:100) overnight at 4 °C. After washing, the membranes were incubated for 2 h with the secondary antibody. The membranes were washed and developed with the West Pico Chemiluminescent substrate (Pierce). Peroxidase activity was detected with a LumiImager device (Roche Applied Science, Mannheim, Germany). The primary antibodies were purchased from Abcam (Cambridge, MA) (GAPDH: Cat.No. Ab9484), Santa Cruz Biotechnology (Santa Cruz, CA) (carbonic anhydrase 3, Cat.No. Sc-50714; cytoglobin, Cat.No. Sc-66855), R&D Systems (Minneapolis, MN) (periostin: Cat. No. AF2955), Sigma-Aldrich (St. Louis, MO) (troponin T: Cat.No. SAB2502131). Western blots were quantified using the Fusioncapt advance software (Vilber Lourmat, Eberhardzell, Germany).

### Cell culture

H9C2 rat cardiac myoblasts were purchased from ATCC (Cat.No. CRL-1446) and cultured in Dulbecco’s modified eagle medium (DMEM; PAN) supplemented with 10 % fetal calf serum (FCS; PAN), 1 % non-essential amino acids, 1 % MEM vitamins, 1 % penicillin/streptomycin at 37 °C in humidified air containing 5 % CO_2_. To mimic ischemia, cell were grown to 70 % confluency, washed three times with PBS and incubated in DMEM serum free supplied with SAR1 or DMSO for 24 h in a special incubation bag containing Anaerocult^®^ A mini (Merck, Darmstadt, Germany), a chemical mixture that completely binds oxygen, creating an oxygen-free environment. The bag was sealed and severe hypoxia (<0.5 % O_2_) was assessed by color change of a test strip (Anaerotest, Merck) from blue to white within the first 90 min of incubation [16, 17]. Human umbilical vein endothelial cells (HUVECs) were kindly provided by Dr. Jennifer Esser. Cells were cultured in enhanced endothelial cell growth medium (PELOBiotech GmbH, Martinsried, Germany) and used for angiogenesis assays.

### Cell-based assays

Caspase-3 activity was determined using a caspase-3 activity assay kit (Enzo Life Sciences, Lörrach, Germany, Cat.No. ALX-260-031). Following the manufacturer’s protocol, cell lysates were incubated with reaction buffer and caspase-3 substrate Ac-DEVD-AMC for 10 min at 37 °C. Fluorescence was measured using a TECAN Infinite M200 microplate reader with excitation and emission wavelengths of 360 and 440 nm, respectively. FACS analysis using PI and YO-PRO^®^-1 (Invitrogen, Cat.No. V13243) was performed on cells plated in 6-well plates at 80,000 cells/well according to manufacturer’s instructions. Fluorescence intensity was measured by flow cytometry (FACScalibur, Fortessa) and evaluated with FlowJo (Tree Star, Inc.). The data were recorded for a total of 15,000 events per sample.

### Statistical analysis

One-way Anova tests were applied separately to compare groups in the mice study. In the rat study, one-way Anova tests were applied to compare infarcted animals under placebo treatment to sham operated animals. Comparison of the different dosages of SAR1 with placebo was done by one-way analysis of variance followed by Tukey’s multiple comparison test. For proteomics and cell-based assays, statistical differences were calculated using Student’s two-sided *t* test. Significance was set to p values <0.05. Data are expressed as mean ± SEM. Statistical analysis was performed using GraphPad version 6.07 for Windows (GraphPad Software, San Diego, CA).

## Results and discussion

### Survival and left ventricular weights upon cathepsin a inhibition in the MI model

**Fig. 1:**
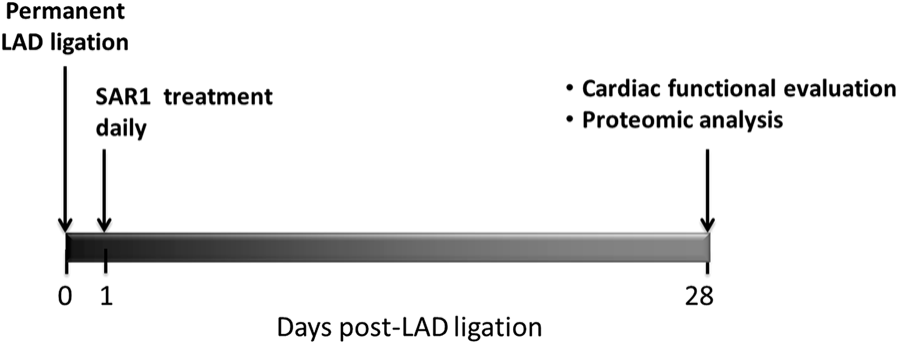
Experimental design of the study. Sham operation or permanent LAD ligation were performed in mice. LAD ligation was either treated with placebo or with the cathepsin A inhibitor SAR1. After 28 days cardiac functionality was evaluated and a quantitative proteome study was conducted

**Fig. 2:**
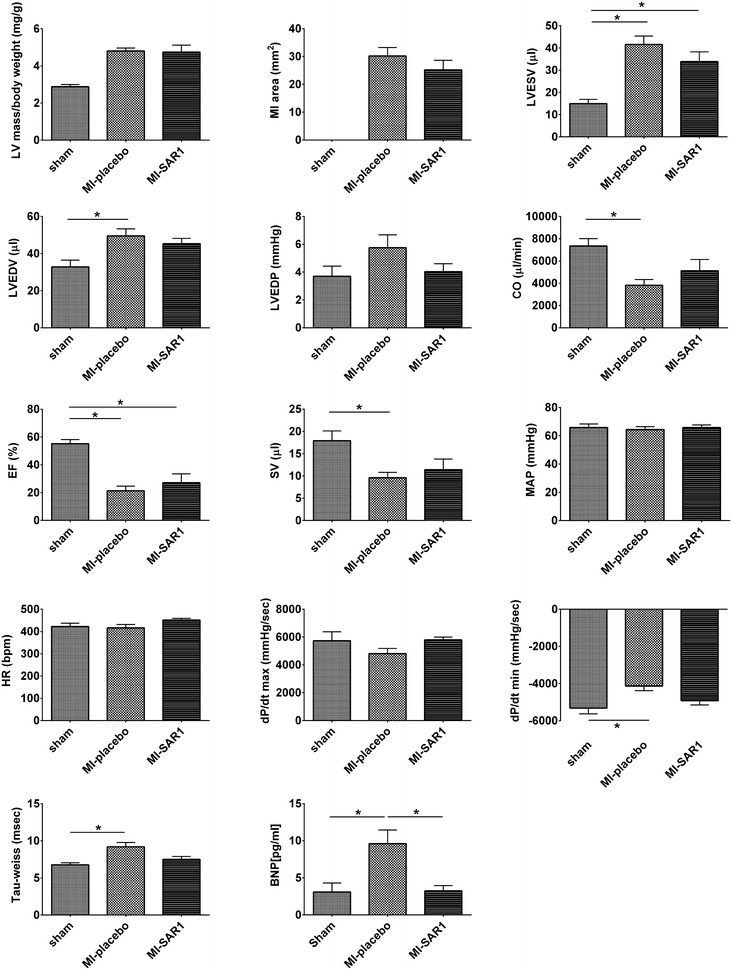
Functional parameters in the murine MI model. Cardiac parameters were assessed 4 weeks after permanent ligation of the LAD. Three groups have been compared: sham (*n* = 10), MI-placebo (*n* = 15), MI-SAR1 (*n* = 10). Results are expressed as mean ± SEM. Differences significant at p < 0.05 are marked with an* asterisk* (*). *LVESV* left ventricular end-systolic volume, *LVEDV* left ventricular end-diastolic volume, *LVEDP* left ventricular end-diastolic pressure, *CO* cardiac output, *EF* ejection fraction, *SV* stroke volume, *MAP* mean arterial pressure, *HR* heart rate, *dP/dt* peak positive or negative first derivative of LV pressure, *BNP* plasma brain natriuretic peptide

To determine the effect of SAR1 on chronic heart failure, we used a mouse model of MI induced by permanent LAD ligation. Three groups were investigated: (1) a sham control group (receiving only a left thoracotomy) (*n* = 10); (2) a placebo-treated MI group (*n* = 15); (3) MI group given the cathepsin A inhibitor SAR1 daily starting one day after operation (100 mg/kg) (*n* = 10) (Fig. 1). Data for the left ventricular (LV) and body weights were determined 4 weeks after operation on the day of hemodynamic measurements. The placebo group showed a significant increase in LV mass/body mass ratio compared to the sham group (Fig. 2), as it is commonly observed in the permanent LAD ligation model of MI [18]. The increase in LV mass/body mass ratio was not affected by SAR1 treatment (Fig. 2). Infarct sizes ranged between 25 and 30 % among mice and were not significantly different between placebo and SAR1-treated animals.

### Cardiac functionality upon cathepsin a inhibition in the MI model

Next, we assessed how cathepsin A inhibition affects cardiac functionality compared to placebo treatment in the murine MI model. As expected, placebo treated animals developed severely impaired cardiac function after 4 weeks of permanent LAD ligation (Fig. 2). These animals displayed hallmark features of post-MI heart failure, including increased left ventricular end systolic volume (LVESV) together with increased left ventricular end diastolic volume (LVEDV) and—pressure (LVEDP). Consequently, cardiac output (CO) and ejection fraction (EF) were severely decreased. Mean arterial pressure (MAP) and heart rate (HR) were not affected by placebo-treated permanent LAD ligation. In line with limited cardiac functionality, dP/dt values were severely impaired; this is also reflected by an increased Tau-weiss value.

Cathepsin A inhibition dampened the increase of LVESV and LVEDP as compared to placebo treatment (Fig. 2), although not meeting statistical significance (One-way Anova test). Its effect on LVEDV was less pronounced. Similarly, cathepsin A inhibition (as compared to placebo treatment) displayed a trend towards a protective effect concerning cardiac output (CO), ejection fraction (EF) and stroke volume (SV). However, the difference between placebo-treated and SAR1-treated animals did not reach statistical significance (One-way anova test). The same picture emerges for dP/dt and Tau-weiss values. In an independent experimental setup, we demonstrated dose-dependent improvement of cardiac functionality by SAR1 in a rat model of permanent LAD ligation (Additional file 1).

Plasma brain natriuretic peptide (BNP) is considered a gold standard biomarker in determining the severity and prognosis of heart failure [19, 20]. We probed BNP plasma levels to monitor the progression of LAD ligation-induced cardiac failure (Fig. 2). Placebo treatment yielded elevated BNP titers post—MI. SAR1 treatment significantly rescued this increase and yielded BNP levels that are within the range of sham-operated animals.

In summary, our data suggest that cathepsin A inhibition by SAR1 may contribute to improved cardiac performance in the rodent permanent LAD ligation model systems.

### Cathepsin A inhibition partially rescues the proteome alterations associated with permanent LAD ligation

In order to further substantiate the promising results yielded by SAR1 treatment of permanent LAD ligation, we used mass-spectrometry-based quantitative proteomics in order to (1) identify proteome alterations associated with the murine permanent LAD ligation and (2) monitor the effect of cathepsin A inhibition on those proteins that are impacted by permanent LAD ligation in the mouse model.

Similar to the section on cardiac functionality, we compared three groups: (1) a sham control group (receiving only a left thoracotomy); (2) a placebo-treated MI group (MI-placebo); (3) MI group given the cathepsin A inhibitor SAR1 daily starting 1 day after operation (100 mg/kg) (MI-SAR1). Left ventricles were harvested 4 weeks after sham operation or LAD ligation. A triple labeling strategy was employed [21] with a total of four biological replicates (12 mice in total).

**Fig. 3:**
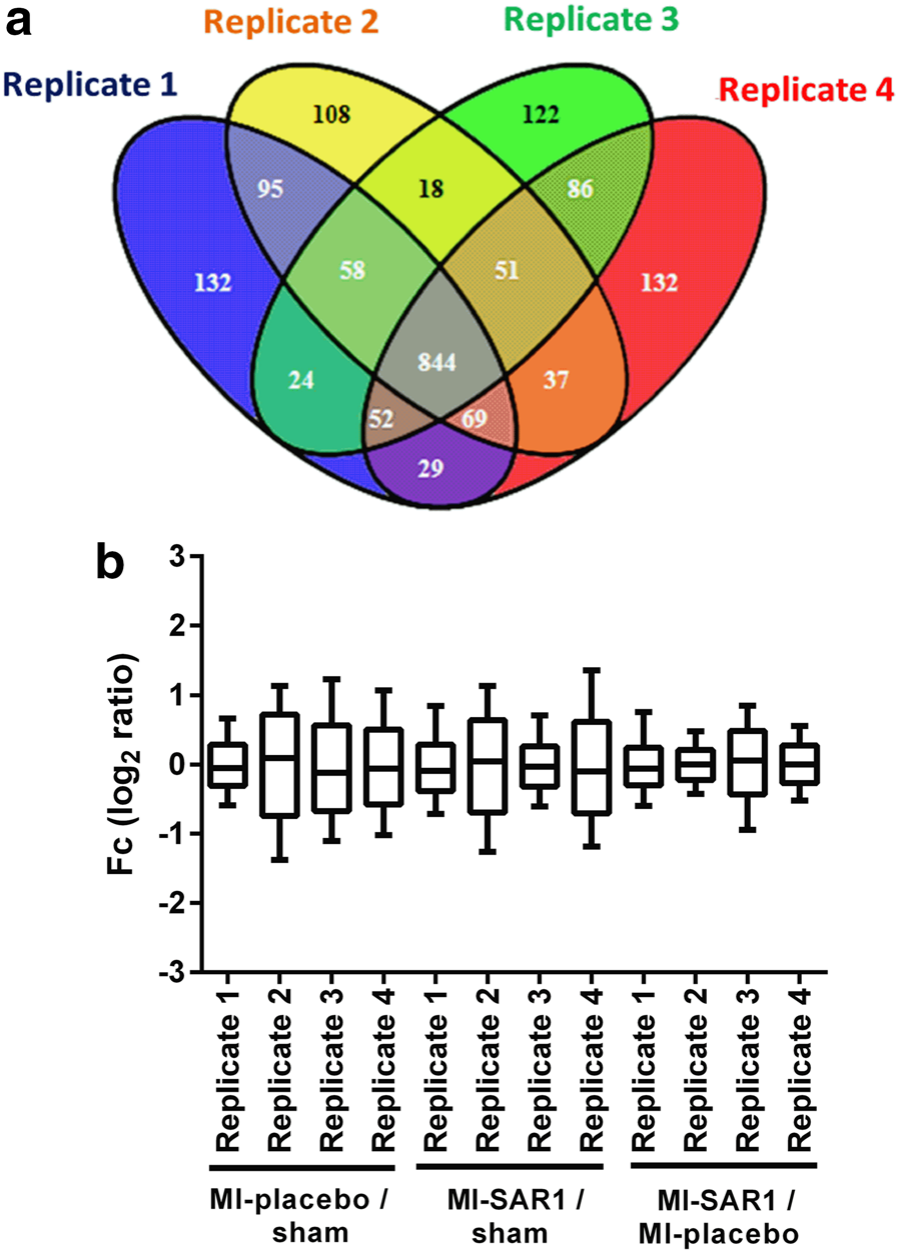
Protein identification and quantification overview for the proteome analysis of murine hearts. **a** *Venn diagram* representing the number of identified proteins in each replicate and their overlap. 1074 proteins were found in at least three replicates. **b** Distribution and geometric mean (*horizontal bar*) of fold change values (log_2_ of relative protein ratios) of proteins from the four biological replicates. Each replicate contains an animal from each group (sham, MI-placebo, MI-SAR1)

In each of the four replicate experiments we identified approximately 1300 proteins (Fig. 3a). 1074 proteins were identified in at least three replicate experiments and we focused on those proteins for further analysis (Additional file 2). As noticed earlier, partially incomplete overlap of proteome coverage is an intrinsic characteristic of mass—spectrometry based proteomics [22]. Protein ratios are expressed as fold-change (Fc) values (log_2_ of light/heavy, light/medium and heavy/medium ratios). All replicates showed a near-normal distribution and an average Fc close to zero, indicating that the majority of the proteins are not altered (Fig. 3b).

We employed the “significance analysis of microarrays” (SAM) method [23] to identify proteins with significantly altered abundance in placebo-treated LAD ligation compared to sham operation. At a false discovery rate <1 % for significantly affected proteins, we identified 79 proteins as increased in placebo-treated LAD ligation and 25 proteins as decreased in placebo-treated LAD ligation, in comparison to sham operation alone. These proteins are summarized in the table included in Additional file 2. This “proteomic fingerprint” of permanent LAD ligation comprises known marker proteins of cardiac failure. For example, we found decreased levels of cardiac troponin T, heart fatty acid-binding protein and α-myosin heavy chain as well as increased levels of atrial natriuretic peptide, galectin-3 and several extracellular/matricellular proteins such as periostin, fibulin-1, versican, lumican and osteoglycin (mimican); thereby being in line with earlier reports [24–26].

Noteworthy, cathepsin A was up-regulated approximately two-fold in placebo-treated permanent LAD ligation compared to sham-treated animals. This increase failed to meet the significance criteria of the SAM method. However, a similar increase of cathepsin A levels was observed in a rat model of chronic heart failure [6]. SAR1 treatment did not change cathepsin A levels in the murine hearts upon LAD ligation (Additional file 3).

**Fig. 4:**
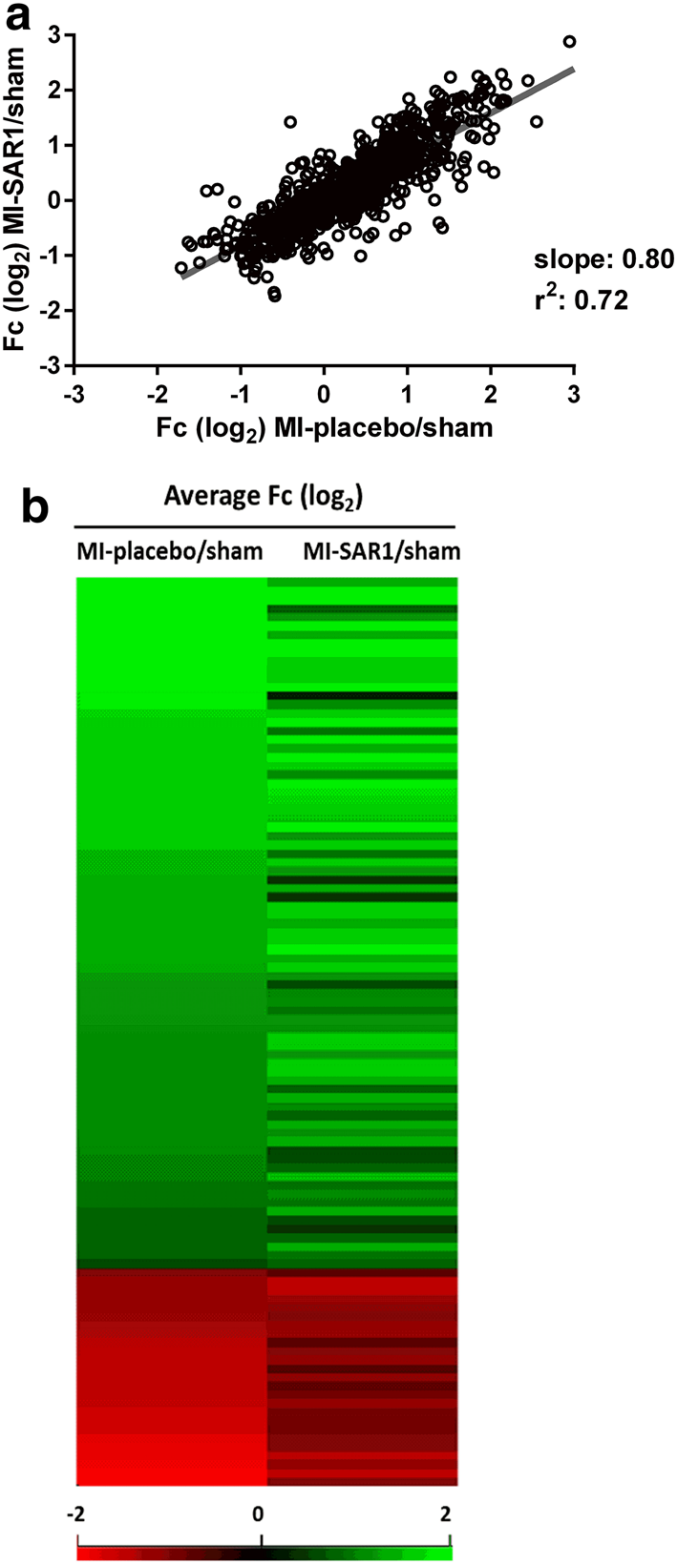
Cardiac proteome fingerprint of cathepsin A inhibition upon LAD ligation. **a** *Scatter plot* and linear regression analysis of protein abundance alterations observed for placebo-treated LAD ligation (as compared to sham operation) and SAR1-treated LAD ligation (as compared to sham operation). **b** Heatmap comparison of fold changes MI-placebo/sham and MI-SAR1/sham of the 104 significantly affected proteins upon LAD ligation. Fold changes in the* first column* (MI-placebo/sham) are placed in descending order

Next, we assessed the effect of SAR1 treatment on the proteomic level. As shown by the scatter plot in Fig. 4a, there is a good correlation between proteome alterations observed upon placebo-treated LAD ligation (as compared to sham operation) and proteome alterations observed upon cathepsin A inhibition (as compared to sham operation). Simple linear regression yields a positive slope of 0.8, indicating that the overall proteome rearrangement upon LAD ligation is similar between placebo and SAR1 treatment but that, on average, the extent of quantitative increase or decrease is mildly dampened upon cathepsin A inhibition.

**Fig. 5:**
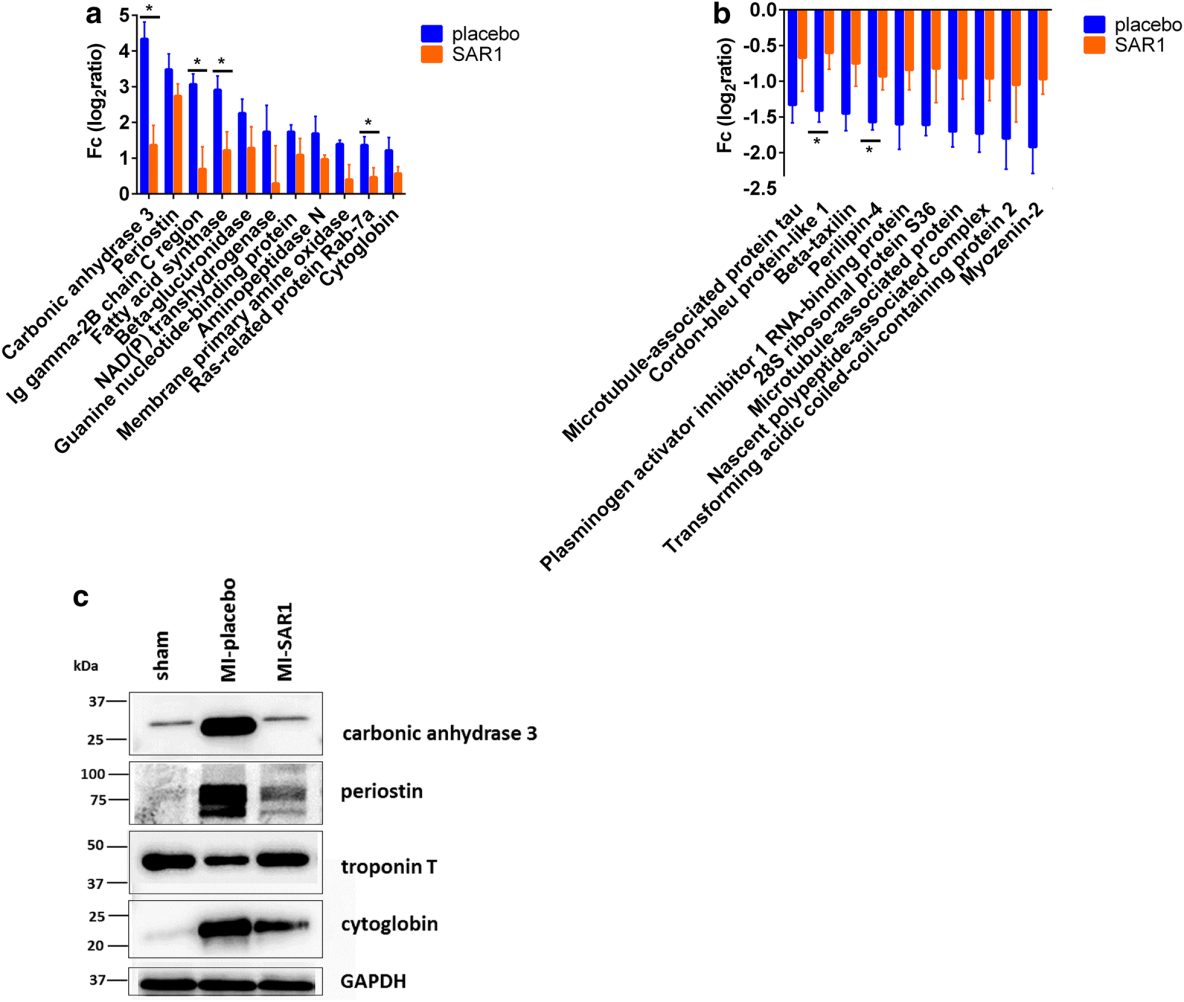
Effect of cathepsin A inhibition on proteome alterations in LAD ligation. **a** *Bar chart* depiction of proteins that are significantly increased in placebo-treated LAD ligation (as compared to sham operation) and for which SAR1 treatment decreased this effect by a fold-change value of more than 50 % [difference of log_2_ ratios (MI-placebo/sham)–(MI-SAR1/sham) > 0.58]. **b** Same as (a) but for significantly decreased proteins in LAD ligation. Results are expressed as mean ± SEM. Differences significant at p < 0.05 are marked with an asterisk (*). **c** Western blot analysis of selected proteins. Shown here is a representative blot. GAPDH is used as loading control

We then focused on those proteins that were initially found to be significantly affected by the placebo-treated LAD ligation (as compared to sham operation). The heatmap in Fig. 4b visualizes the effect of SAR1 on this subgroup. It becomes evident that SAR1 treatment partially restores the ligation-induced alterations for many proteins in this subgroup. For a more detailed view on proteins for which SAR1 rescues, at least in part, a ligation-induced alteration, we concentrated on those proteins that were (1) significantly affected by placebo-treated LAD ligation (as compared to sham operation) and (2) for which SAR1 treatment increased or decreased this effect by more than 50 % (based on averaged protein ratios from the replicate experiments; difference of log_2_ ratios (MI-placebo/sham)– (MI-SAR1/sham) >0.58). These criteria were fulfilled by 21 proteins (Fig. 5a, b). In every case, SAR1 treatment dampened the alteration in protein abundance that was caused by LAD ligation (as compared to sham operation). This effect reached statistical significance (p < 0.05, two-sided t-test) for carbonic anhydrase 3, Igγ 2b, fatty acid synthase, and Rab-7a (attenuated increase in abundance, Fig. 5a) as well as cordon-bleu protein-like 1 and perilipin-4 (attenuated decrease in abundance, Fig. 5b).

Carbonic anhydrase 3 (CA3) is one of the most up-regulated proteins upon LAD ligation (~nine-fold increase compared to sham operation). Elevated plasma levels of CA3 in MI have been previously reported [27, 28]. Elevated plasma levels of CA3 are also found in Duchenne muscular dystrophy [29]. These findings suggest that increased levels of CA3 are associated with degenerating or stressed muscle tissue. SAR1 treatment rescued elevated CA3 levels caused by LAD ligation. This was further validated by western blot analysis (Fig. 5c).

FAS is a biosynthetic enzyme responsible for de novo fatty acid synthesis in mammals. FAS was found to be increased in two mechanistically distinct mouse models of heart failure and in the hearts of humans with end stage cardiomyopathy [30]. We find a more than six-fold increase of FAS abundance upon placebo-treated LAD ligation (as compared to sham operation). In line with an increased need for de novo fatty acid synthesis, we also noticed reduced levels of perilipin-4, which is required for intracellular storage of lipid droplets. In both cases, cathepsin A inhibition significantly dampened the altered abundance observed upon placebo-treated LAD ligation (as compared to sham operation, Fig. 5a, b).

Interestingly, we observed that cathepsin A inhibition also attenuates elevated levels of cardiac stress response proteins, such as periostin, troponin T and cytoglobin. It was recently found that cytoglobin expression is strongly up-regulated in the hypoxia-induced hypertrophic heart [31]. We corroborated the proteomic results observed for periostin, troponin T and cytoglobin by immunoblot analysis (Fig. 5c; Additional file 4).

In summary, our proteomic results indicate that SAR1 treatment reduces the extent of cardiac proteome rearrangement in permanent LAD ligation.

### Effect of cathepsin A inhibition in an in vitro ischemia model

**Fig. 6:**
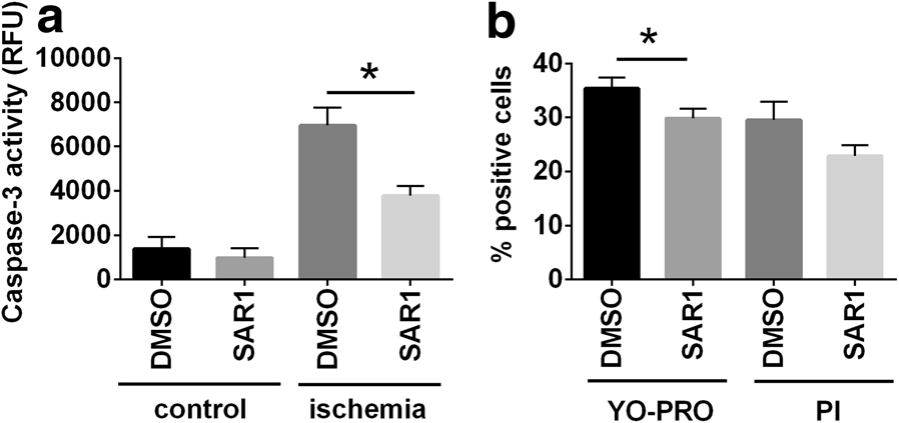
Effect of cathepsin A inhibition in an in vitro model of ischemia. Cells were incubated for 24 h with 10 μM SAR1 or DMSO as a solvent-only control in simulated ischemia culture conditions (hypoxia and serum deprivation, see Methods section for details). **a** Quantitative analysis of caspase-3 activity in the total cell lysate of cells treated with SAR1 in standard conditions (normoxia; DMEM supplemented with FCS) and simulated ischemia (hypoxia; DMEM serum-free). **b** Cells in simulated ischemia were stained with YO-PRO-1 or PI and evaluated by flow cytometry. The percentage of cells positive to YO-PRO-1 or to PI was calculated. Results are expressed as mean ± SEM. Differences significant at p < 0.05 are marked with an* asterisk *(*)

The rat cardiomyocyte cell line H9C2 has been previously used in cell models of heart ischemia and ischemia–reperfusion [32, 33]. In order to probe a putative cardio-protective effect of SAR1 in a cell-autonomous system, we subjected H9C2 cells to culture conditions of simulated ischemia, which consist of serum deprivation and severe hypoxia. After 24 h, we assessed caspase-3 activity as a marker of apoptosis. SAR1 treatment significantly reduced the induction of caspase-3 activity elicited by simulated ischemia (Fig. 6a). Flow-cytometry of cells stained for the apoptotic marker YO-PRO-1 and the necrosis marker PI showed that SAR1 treatment during simulated ischemia significantly reduced the percentage of cells undergoing apoptosis and, to a lesser extent, necrosis (Fig. 6b). Our findings suggest that cathepsin A inhibition attenuates ischemia-induced apoptosis in H9C2 cells. Additionally, we excluded a hypothetical effect of SAR1 on angiogenesis by a matrigel capillary-like sprouting assay using HUVECs treated with SAR1 for 16 h (Additional file 5).

## Conclusions

The present study suggests that cathepsin A inhibition attenuates the impact of MI on the functional and proteomic levels, as probed in a rodent model of permanent LAD ligation. For most functional parameters, the effect of SAR1 remained a protective trend since statistical significance was not fully reached. However, cathepsin A inhibition significantly rescued LAD ligation induced alterations in abundance for several proteins. Examples include plasma BNP and tissue-resident CA3. Since initial phase 1 studies indicate a favorable safety profile for SAR1 [9], our results encourage further investigation in cathepsin A inhibition for the treatment of post MI heart failure.

## Additional files

10.1186/s12967-016-0907-8 SAR1 treatment upon LAD permanent ligation in rats. A) Experimental design of the study performed in a rat model. Sham operation or permanent LAD ligation were performed in rats. LAD ligation was either treated with placebo or with the cathepsin A inhibitor SAR1 at different doses (3, 10 and 30 mg/kg, p.o.). After 7 days cardiac functionality was evaluated. B) Hemodynamic data from rats after one week of permanent LAD ligation. LVEDP, left ventricular end-diastolic pressure; LVP, left ventricular pressure; HR, heart rate; dP/dt, peak positive or negative first derivative of LV pressure. Results are expressed as mean ± SEM. Differences significant at p < 0.05 are marked with an asterisk (*).
10.1186/s12967-016-0907-8 Overview of proteins that were identified and quantified in at least three of the four replicate experiments.
10.1186/s12967-016-0907-8 Cathepsin A levels upon LAD ligation and SAR1 treatment Average fold change values (log_2_ of relative protein ratios) of cathepsin A in the three experimental groups.
10.1186/s12967-016-0907-8 Densitometric analysis of troponin T, periostin, carbonic anhydrase 3 and cytoglobin in sham, MI-placebo and MI-SAR1 mice. The densitometric measurements of proteins are expressed as a percentage of the average values measured in the sham group. Results are expressed as mean ± SEM.
10.1186/s12967-016-0907-8 Angiogenesis assay-Matrigel capillary-like sprouting assay. Serum-starved human umbilical venous endothelial cells (HUVECs) were untreated or treated with DMSO, or SAR1 10 μM, or with vascular endothelial growth factor (VEGF; 50 ng/mL) for 16–18 h before they were seeded onto Matrigel (Corning BV, Amsterdam, The Netherlands, Cat.No. 356237). Duplets of 2 × 10^4^ cells per condition were cultured on phenol red-free Matrigel with 2 % FBS for 3 h at 37 °C, 5 % CO_2_. Cells were fixed with 4 % paraformaldehyde (PFA) and pictures were taken from four random microscopic fields at 5× magnification using a digitized imaging system. The cumulative sprout length and the number of branch points were measured with AxioVision Rel. 4.8.

## Abbreviations

MI: myocardial infarction
LAD: left anterior descending artery
LVESV: left ventricular end systolic volume
LVEDV: left ventricular end diastolic volume
LVEDP: left ventricular end diastolic pressure
CO: cardiac output
EF: ejection fraction
MAP: mean arterial pressure
HR: heart rate
SV: stroke volume
BNP: brain natriuretic peptide
SAM: significance analysis of microarrays
CA3: carbonic anhydrase 3
FAS: fatty acid synthase
PI: propidium iodide
